# Supplementary material for: Nebulized fusion inhibitory peptide protects cynomolgus macaques from measles virus infection
Source: Res Sq. 2022 Jun 1:rs.3.rs-1700877. Preprint. [Version 1] doi: 10.21203/rs.3.rs-1700877/v1 (PMC9176655; doi:10.21203/rs.3.rs-1700877/v1)
Supplement: Supplement 1 [file Reynard-Supplementary.pdf]

Supplementary Materials

Nebulized fusion inhibitory peptide protects cynomolgus macaques from measles virus infection

Olivier Reynard[1], Claudia Gonzalez[1], Claire Dumont[1], Mathieu Iampietro[1], Marion Ferren[1], Sandrine Le Guellec[2], Lajoie Laurie[3], Cyrille Mathieu[1], Gabrielle Carpentier[4], Georges Roseau[4], Francesca T. Bovier[6], Yun Zhu[6,7], Deborah Le Pennec[5], Jérome Montharu[4], Amin Addetia[8], Alexander L. Greninger[8], Christopher A. Alabi[9], Anne Moscona[6,10,11], Laurent Vecellio[4], Matteo Porotto[6,12], Branka Horvat[1]

- 2 supplementary tables

- 6 Supplementary figures

Table S1. Performance of nebulizers used in the study, loaded with 3 ml of either saline or peptide.

Table S2 Deposition of the aerosol in organs of nebulized animals

Fig. S1. Evaluation of HRC4 dose and treatment schedule in CD150xIFNα/β R KO murine model of MeV infection.

Fig. S2. Distribution in nebulized HRC4 peptide in lungs of cynomolgus monkeys.

Fig. S3. Histopathological analysis of lungs from HRC4-nebulised cynomolgus macaques.

Fig. S4. **Schematic presentation of the peptide and virus deposition in the cell culture model.**

Fig. S5. **Evolution of the hematological parameters during the MeV infection.**

Fig. S6. **Evolution of major PBMC populations in blood of cynomolgus macaques.**

Table S1. Performance of nebulizers used in the study, loaded with 3 ml of either saline or peptide and presented as mean ± SD of four tested devices.

	[1]VMD (µm)	[2]< 5µm (%)	[2]< 2µm (%)	[3]Duration (min)	[4]Output rate (ml/min)	[5]Residual volume (ml)	[6]Output (%)
NaCl (0.9 %)	4.37 +/- 0.16	59.8 +/- 2,8	8.5 +/- 0.8	6.9 +/- 1.9	0.46 +/- 0.13	0.05 +/- 0.01	98.5 +/- 0.4
HRC4 (4 mg/ml)	4.43 +/- 0.26	57.4 +/- 3.9	13.9 +/- 2.0	9.3 +/- 1.2	0.32 +/- 0.04	0.06 +/- 0.04	98.1 +/- 1.3

[1] VMD for Volume Median Diameter, presents the mean size of generated aerosols, measured by laser diffraction method (Spraytec Malvern Instrument)

[2] Percentage of particles smaller than 5 µm or 2 µm, presents the fraction of aerosol below the indicated size, corresponding to the aerosol penetrating into either lungs in general (< 5µm) or into alveolar regions of lungs (< 2µm).

[3] Duration corresponds to the time necessary to achieve complete aerosolization of the 3 ml loaded solution

[4] Output rate presents the aerosol flow rate in ml per minute

[5] Residual volume corresponds to the volume of liquid remaining in the reservoir at the end of the nebulization

[6] Output corresponds to the percentage of liquid volume delivered by the nebulizer compared to the initial charge of each nebulizer (n = 4).

Table S2. Deposition of the aerosol in organs of nebulized animals.

***Deposition in macaque (%)**	**Animal 1.1**	**Animal 1.2**	**Animal 2.1**	**Animal 2.2**	**Mean +/- SD**
Lungs	8.0	11.3	15.5	10.7	**11.4 +/- 3.1**
Upper respiratory Tract	15.5	28.0	44.4	30.3	**29.5 +/- 11.9**
Stomach	15.7	22.0	0.4	9.7	**11.9 +/-9.2**
Esophagus / trachea	1.2	1.7	1.1	9.7	**3.4 +/- 4.2**

* ^{99m}TC-DTPA (74 MBq) was administrated in 3 ml NaCl 0.9% by prototype mesh nebulizer in four experiments (2 different animals were nebulized independently twice). The deposition was analyzed by E-cam gamma camera and calculated from the generated digitalized images.

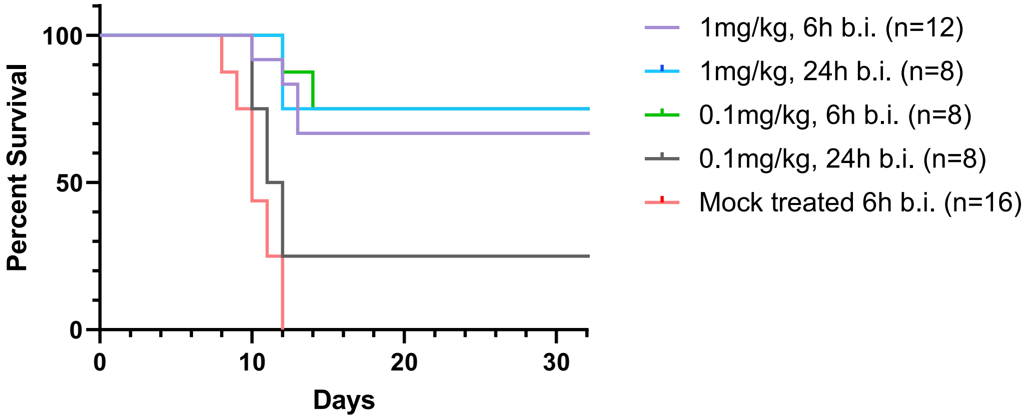

Fig. S1. Evaluation of HRC4 dose and treatment schedule in CD150xIFNa/bR KO murine model of MeV infection. Mice were pretreated intranasally either 6 h or 24 h before infection (b.i.) with the indicated dose of HRC4 and inoculated with 10^4 PFU of MeV IC323. HRC4 lipopeptide at 1 mg/kg efficiently protected CD150xIFNa/bR KO mice from intranasal MeV infection when given 6 h and 24 h before infection ($p < 0.0001$, Mantel-Cox test)

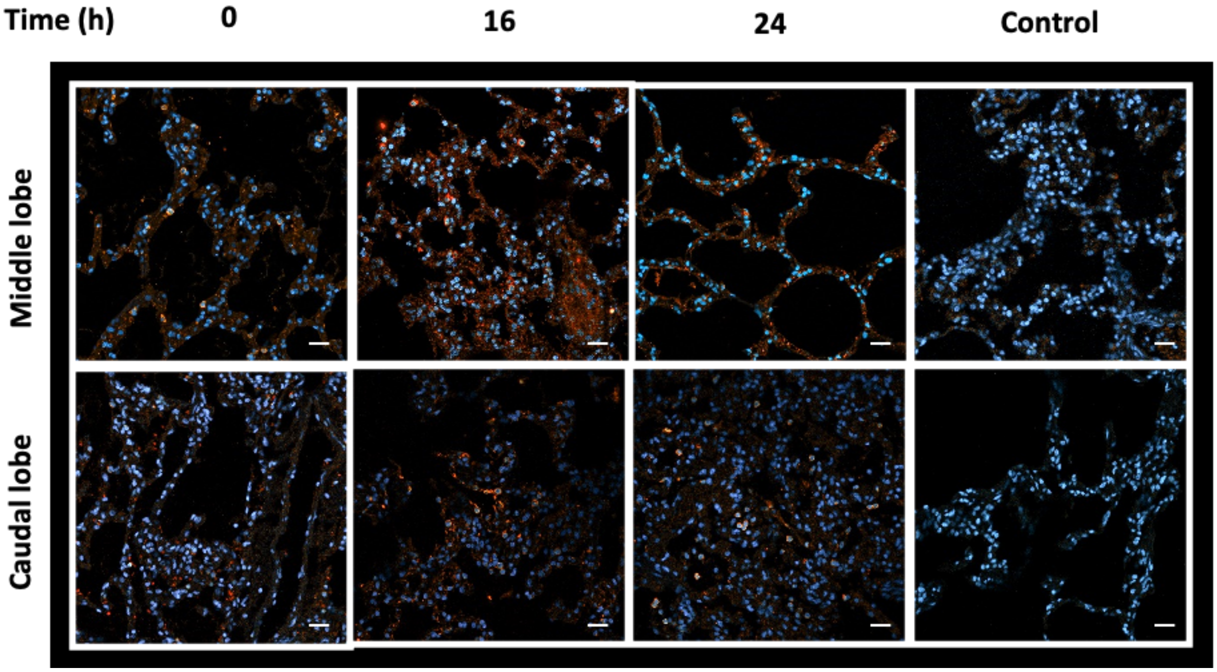

Fig. 2. Distribution in nebulized HRC4 peptide in lungs of cynomolgus monkeys. HRC4 nebulization was made at indicated time points: 15 min (T0), 16 h (T16) or 2 4h (T24) under mechanical ventilation prior to euthanasia and control NHP did not receive peptide. Peptide biodistribution was assessed in caudal (low), middle (medium) and cranial lobes (upper) (Fig. 3B), of the right lung. Paraffin embedded lung sections from NHPs were analyzed by the immunofluorescence, using rabbit anti-HRC peptide and goat anti-rabbit Alexa 555 (orange staining) and DAPI was used to stain nuclei (blue staining), and Imaging was done using a Zeiss LSM800 at 20x.

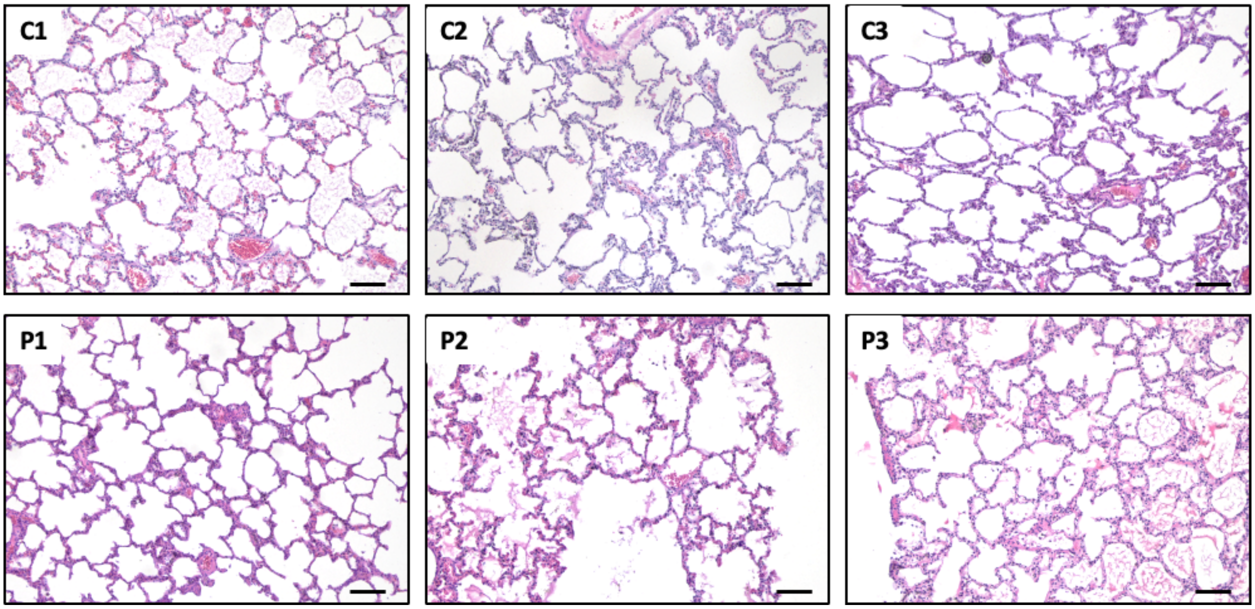

Fig. S3. Histopathological analysis of lungs from HRC4-nebulised cynomolgus macaques. Animals were nebulized 15 min (C1, C2, C3), 16 h (P2) or 24 h (P1, P3) prior to euthanasia and lung sections from paraffin embedded organs from nebulized were stained with hematoxylin and eosin. Analysis of multiples lung sections form each animal presented preserved lung histology and absence of any visible adverse effects**.** Images were taken on a Nikon TS2r microscope at 10x and scale bars correspond to 200 μM.

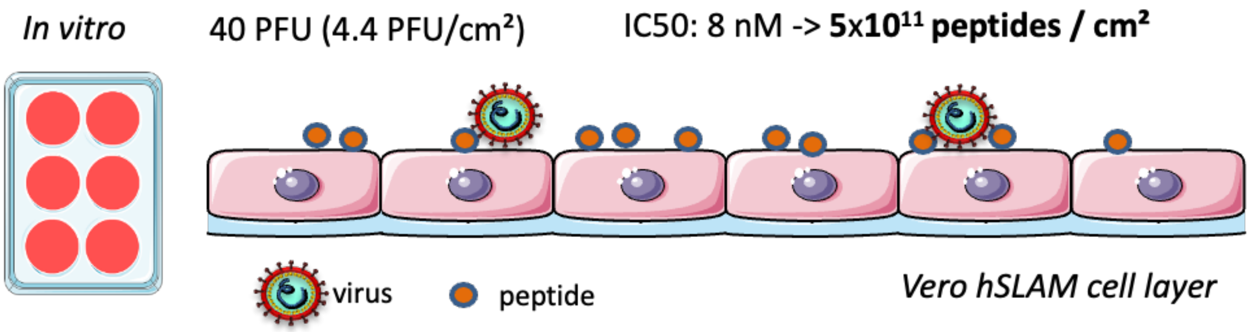

Fig. S4. **Schematic presentation of the peptide and virus deposition in the cell culture model.** Peptide and virus deposition in the *in vitro* assays, presented in the Fig. 1E, performed in 6 well plates (9 cm^2/well), using 40 PFU of MeV/well, was calculated giving a ratio of 4.4 PFU/cm^2. Dose response experiments indicated an IC_{50} of 8 nM, which correspond to a total of 4.81x10^{12} molecules of peptides per well and a ratio to surface of 5x10^{11} peptides/cm^2, presenting the amount of peptide very close to the one deposited in macaques' lung (Fig. 5).

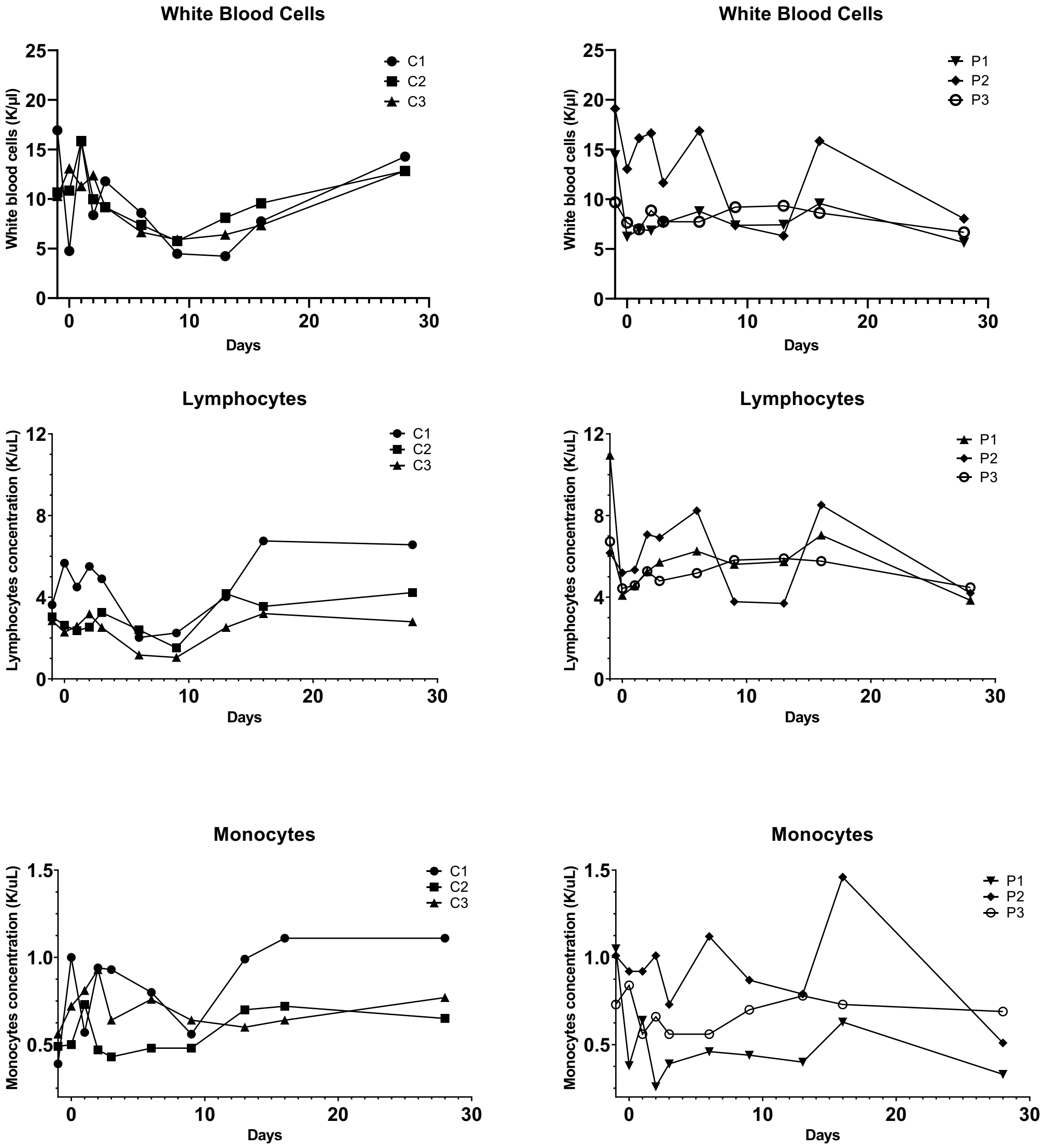

Fig. S5. **Evolution of the hematological parameters during the MeV infection.** Major PBMC populations in blood of cynomolgus macaques were followed using Sysmex XT2000i Vet automatic analyzer, after nebulization of either 3 ml of 0.9% NaCl (C) or 4 mg/kg of HRC4 peptide (P) and MeV infection. Transient lymphopenia is observed in the absence of peptide protection.

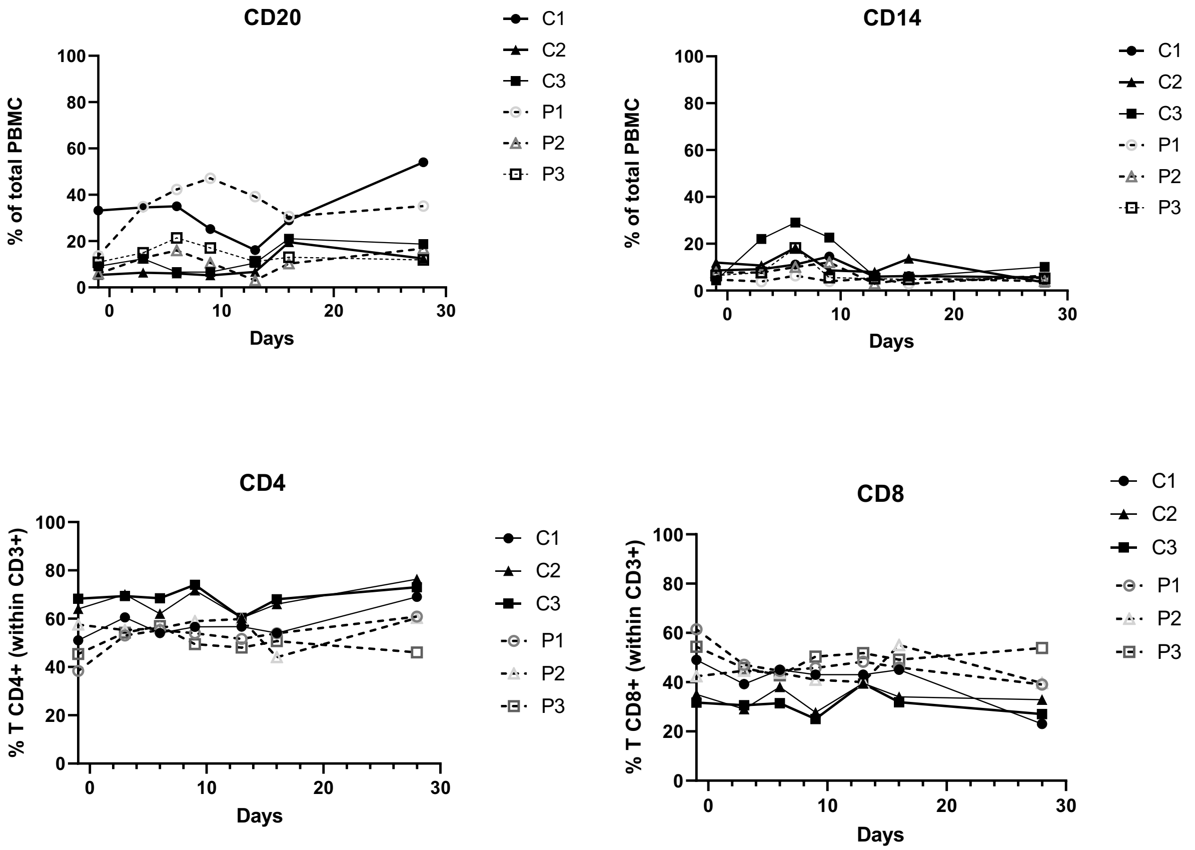

Fig. S6. **Evolution of major PBMC populations in blood of cynomolgus macaques.** Following nebulization of either 0.9% NaCl (C) or 4 mg/kg of HRC4 peptide (P) and MeV infection, indicated cell populations were followed in the blood of animals at indicated time points, using a MACSQuant® 10 flow cytometer (Miltenyi).
